# Presence and activation of pro-inflammatory macrophages are associated with CRYAB expression in vitro and after peripheral nerve injury

**DOI:** 10.1186/s12974-021-02108-z

**Published:** 2021-03-24

**Authors:** Erin-Mai F. Lim, Vahid Hoghooghi, Kathleen M. Hagen, Kunal Kapoor, Ariana Frederick, Trisha M. Finlay, Shalina S. Ousman

**Affiliations:** 1 Department of Neuroscience, Hotchkiss Brain Institute, University of Calgary, 3330 Hospital Drive N.W., Heritage Medical Research Building, Calgary, Alberta T2N 4N1 Canada; 2 Department of Clinical Neurosciences, Hotchkiss Brain Institute, University of Calgary, 3330 Hospital Drive N.W., Heritage Medical Research Building, Calgary, Alberta T2N 4N1 Canada; 3 Department of Cell Biology & Anatomy, Hotchkiss Brain Institute, University of Calgary, 3330 Hospital Drive N.W., Heritage Medical Research Building, Calgary, Alberta T2N 4N1 Canada

**Keywords:** Peripheral nervous system, Peripheral nerve injury, Macrophages, Pro-inflammatory, CRYAB, AlphaB-crystallin, Cytokines, Chemokines

## Abstract

**Background:**

Inflammation constitutes both positive and negative aspects to recovery following peripheral nerve injury. Following damage to the peripheral nervous system (PNS), immune cells such as macrophages play a beneficial role in creating a supportive environment for regrowing axons by phagocytosing myelin and axonal debris. However, a prolonged inflammatory response after peripheral nerve injury has been implicated in the pathogenesis of negative symptoms like neuropathic pain. Therefore, the post-injury inflammation must be carefully controlled to prevent secondary damage while allowing for regeneration. CRYAB (also known as alphaB-crystallin/HSPB5) is a small heat shock protein that has many protective functions including an immunomodulatory role in mouse models of multiple sclerosis, spinal cord injury, and stroke. Because its expression wanes and rebounds in the early and late periods respectively after PNS damage, and CRYAB null mice with sciatic nerve crush injury display symptoms of pain, we investigated whether CRYAB is involved in the immune response following PNS injury.

**Methods:**

Sciatic nerve crush injuries were performed in age-matched *Cryab* knockout (*Cryab*^*−/−*^) and wildtype (WT) female mice. Nerve segments distal to the injury site were processed by immunohistochemistry for macrophages and myelin while protein lysates of the nerves were analyzed for cytokines and chemokines using Luminex and enzyme-linked immunosorbent assay (ELISA). Peritoneal macrophages from the two genotypes were also cultured and polarized into pro-inflammatory or anti-inflammatory phenotypes where their supernatants were analyzed for cytokines and chemokines by ELISA and protein lysates for macrophage antigen presenting markers using western blotting.

**Results:**

We report that (1) more pro-inflammatory CD16/32^+^ macrophages are present in the nerves of *Cryab*^*−/−*^ mice at days 14 and 21 after sciatic nerve crush-injury compared to WT counterparts, and (2) CRYAB has an immunosuppressive effect on cytokine secretion [interleukin (IL)-β, IL-6, IL-12p40, tumor necrosis factor (TNF)-α] from pro-inflammatory macrophages in vitro.

**Conclusions:**

CRYAB may play a role in curbing the potentially detrimental pro-inflammatory macrophage response during the late stages of peripheral nerve regeneration.

**Supplementary Information:**

The online version contains supplementary material available at 10.1186/s12974-021-02108-z.

## Background

Hematogenously-derived macrophages are essential for successful regeneration of damaged peripheral nervous system (PNS) axons. Due to the secretion of cytokines and chemokines such as monocyte chemoattractant protein-1 (MCP-1), tumor necrosis factor alpha (TNF-α), interleukin (IL) 1 beta (IL-1β), interleukin 6 (IL-6), and leukemia inhibitory factor (LIF) by de-differentiated Schwann cells following peripheral nerve injury, monocytes are recruited from the blood compartment into the distal segment of damaged axons within 2–3 days post-injury [1–4]. These monocytes differentiate into macrophages that produce additional TNF-α, IL-6, IL-1-α, and IL-1β that contribute to further infiltration of blood-derived monocytes [3–7]. The mature macrophages, along with de-differentiated Schwann cells and proliferating resident macrophages, play a central role in creating a supportive environment for regenerating axons because they secrete neurotrophins and phagocytose and clear axonal and myelin debris that contain inhibitors against neurite growth. Indeed, their delayed recruitment or depletion results in reduced myelin debris clearance, loss of neurotrophin synthesis, and compromised axonal regeneration and functional recovery [1, 8–10]. However, a prolonged inflammatory response has been implicated in the pathogenesis of negative symptoms after peripheral nerve injury or other nerve diseases [11, 12]. For example, prolonged presence of IL-1β and TNF-α can promote cell death and neurodegeneration as well as mediate neuroma formation by stimulating fibroblast proliferation [7, 12]. Further, macrophages have a crucial role in the development of peripheral nerve injury-induced neuropathic pain [13–18]. It is thus important to understand how the beneficial properties of macrophages can be harnessed while reducing their pathological functions after peripheral nerve injury.

Macrophages are divided into different phenotypes according to their function or stimulation. In terms of function, these immune cells can be broadly classified as pro-inflammatory (generally called M1) or anti-inflammatory (M2). Anti-inflammatory phenotypes can be further divided into subpopulations due to their diverse modes of stimulation: M2a (activated by IL-4 and IL-13), M2b (activated by immune complexes in combination with IL-1β and lipopolysaccharide (LPS)), and M2c (activated by IL-10, transforming growth factor beta (TGF-β), or glucocorticoids) [19]. It is now recognized that the phenotype of macrophages is much more complex than M1 and M2 and that the cells exist as a continuum of states that shift back and forth depending on the type of stimuli present in the environment. Indeed, this has also been shown for peripheral nerves [20, 21]. We will thus refer to macrophages as either pro- or anti-inflammatory. What is incompletely known are the factors that control the various functional states of macrophages in peripheral nerves after damage.

CRYAB (also known as HSPB5 or alphaB-crystallin) is a small heat shock protein that has been shown to possess a number of protective functions including chaperoning [22–24], pro-survival [25–28], anti-inflammatory [29], remyelinating [30], and anti-neurotoxic [31] abilities. With respect to its immunomodulatory role, treatment with recombinant human CRYAB was demonstrated to dampen the inflammatory response in injured spinal cord [32] while mice that are null for the heat shock protein display enhanced T cell and macrophage activation in experimental allergic encephalomyelitis [33–35] and stroke [36, 37]. Because of its expression in PNS axons and Schwann cells [30, 38–40], altered levels post-PNS damage [30] (reduced and then increased) and enhanced symptoms of pain in *Cryab*^*−/−*^ mice with sciatic nerve crush injury [30], we investigated whether the small heat shock protein is involved in macrophage infiltration and phenotype following peripheral nerve injury. We found that the presence of macrophages is prolonged in sciatic nerves after damage in *Cryab* null mice and that these immune cells were primarily of a pro-inflammatory nature. Further, in vitro experiments with LPS-activated peritoneal macrophages suggest that the heat shock protein may contribute to inhibiting pro-inflammatory cytokine production by macrophages.

## Methods

### Mice

*Cryab-*null (*Cryab*^*−/−*^) mice were developed at the National Institutes of Health National Eye Institute [41]. The animals were generated from embryonic stem cells with a 129S4/SvJae background and maintained on a 129S6/SvEvTac X 129S4/SvJae background. *Cryab*^*−/−*^ mice are viable and fertile. At approximately 40 weeks of age, these animals display a curved spinal cord and degeneration of muscles [41]. We studied these animals between 8 and 12 weeks of age to ensure that the aging characteristics did not confound our results. This was validated by performing analyses on age-matched uninjured 129S6/SvEvTac wildtype (WT) and *Cryab*^*−/−*^ mice before injury to confirm equivalent baseline properties. Colonies of WT and *Cryab*^*−/−*^ mice were bred and maintained in our animal facility where they had access to food and water ad libitum. All procedures were carried out in accordance with guidelines of the Canadian Council of Animal Care and had received approval by the University of Calgary Animal Resources and Ethics Committee.

### Surgery

Eight to 12-week-old female WT and *Cryab*^*−/−*^ mice were anesthetized with a 3:1 ketamine/xylazine (200 mg/kg:10 mg/kg) mixture by intraperitoneal injection. An incision was made through the skin below the hip, and the muscle was blunt dissected using fine surgical scissors and forceps to expose the right sciatic nerve at mid-thigh level. The nerve was crushed 0.5 cm above the region where it splits into the sural, common peroneal, and tibial branches. The sciatic nerve was crushed using 5.0 fine forceps for 30 s; after which, the forceps were rotated 90°, and the same area was crushed for an additional 30 s to ensure that the majority of axons were crushed [30]. Animals were allowed to recover on a heated pad, and their sciatic nerves were harvested at 1, 3, 5, 7, 14, 21, or 28 days post-injury.

### Immunohistochemistry

Naïve and post-crushed mice were euthanized with carbon dioxide. A 5-mm segment of the sciatic nerve starting at 3 mm from the sciatic notch area was removed from naïve animals while a 5-mm portion of the nerve 3 mm distal from the crush site was obtained from injured animals. Nerves were fixed in Zamboni’s fixative for 2 h at room temperature, cryoprotected in 30% sucrose solution for 48 h, embedded into optimum cutting temperature compound, and frozen. Ten-micrometer thick cross-sections were obtained where sectioning was initiated at the end of the dissected nerve segment that was 3 mm distal to the crush site. Sections were then blocked with phosphate-buffered saline containing 0.1–0.3% Triton X-100 and 5–10% normal goat serum for 1 h at room temperature or 3 days at 4 °C (iNOS and Arg1). The tissues were incubated overnight at 4 °C with rabbit anti-Iba1 (Wako Chemicals, 019-1974, 1:200) or goat anti-Iba1 (Novus Biologicals, NB100-1028, 1:100), rat anti-mouse CD16/32 (BD Biosciences, 553842, 1:500), goat anti-mouse CD206 (Santa Cruz Biotechnologies, sc-34577, 1:500), chicken anti-P0 (Abcam, ab39375, 1:500), rabbit anti-CD68 (Cell Signaling, 97778, 1:100), rabbit anti-arginase 1 (Cell Signaling, 93668, 1:50), rabbit anti-iNOS (BD Transduction Laboratories, 1:100; gift from Dr. Samuel David, McGill University), and DAPI (Invitrogen, D3571, 1:2000). Bound antibody was detected using the Invitrogen secondary antibodies, anti-rabbit 488 (A11008), anti-rabbit 594 (A21207), anti-rat 488 (A11006), or anti-goat 488 (A11055) at 1:200 or 1:500.

#### Immunohistochemistry quantification

Three to four cross-sections that were 50 μm apart from each other were obtained from every animal in both genotypes. Following immunostaining, the whole cross-sectional-stained area of the sciatic nerve was obtained at × 20 magnification with an Olympus Slide Scanner microscope. The number of Iba1^+^ DAPI^+^, CD206^+^ Iba1^+^ DAPI^+^, CD16/32^+^ Iba1^+^ DAPI^+^, and P0^+^ Iba1^+^ DAPI^+^ profiles was quantified in the entire sciatic nerve cross-sectional area using Olympus cellSens. Each z-series (depth of x μm) was processed into a single focused image using extended focal imaging. A region of interest was drawn around the sections and a minimum object size of 150 pixels was defined for measurements. A manual global intensity threshold was determined on random images for each of the three channels, and all images were segmented using these same thresholds. The engulfing of myelin debris by macrophages was identified by the colocalization of overlapping pixels in the segmented images. Object counts, areas, and area fractions were obtained for each group.

### Luminex

A 5-mm segment of the sciatic nerve starting from the sciatic notch area was removed from naïve animals while a 5-mm portion of the nerve 3 mm distal from the crush site was obtained from injured animals at various time points post-crush (1, 3, 5, 7, 14, 21, and 28 days) from WT and *Cryab*^−/−^ mice (*n* = 3–4/group) and frozen. The nerves were then homogenized in a solution containing 50 mM Tris-hydrochloride (Tris-HCL) pH 7.4, 1% NP-40, 10% glycerol, 1 mM ethylenediaminetetraacetic (EDTA), 1 mM sodium vanadate (Na_3_VO_4_), 1 mM sodium fluoride (NaF), 1 mM dithiothreitol (DTT), 4.5 mM sodium pyrophosphate, 10 mM β-glycerophosphate, and a protease inhibitor cocktail tablet (Roche Diagnostics). The supernatants were collected after centrifugation at 14,000 rpm at 4 °C for 20 min, and protein content was determined with a spectrophotometer using absorption at 570 nm. All samples were diluted to 667 μg/mL to ensure equal amounts of protein. The protein levels of 31 cytokines and chemokines were assayed using Eve Technologies’ (Calgary, AB, Canada) Mouse Cytokine Array/Chemokine Array 31-Plex laser bead technology (MD31) and results analyzed using Bead Analyzer (Bio-Plex 200). The array factors were as follows: eotaxin, granulocyte-colony stimulating factor (G-CSF), granulocyte-macrophage colony-stimulating factor (GM-CSF), IL-1α, IL-1β, IL-2, IL-3, IL-4, IL-5, IL-6, IL-7, IL-9, IL-10, IL-12p40, IL-12p70, IL-13, IL-15, IL-17A, interferon gamma (IFN)-γ, interferon gamma-induced protein 10 (IP-10), keratinocyte chemoattractant (KC), LIF, lipopolysaccharide-inducible CXC chemokine (LIX), macrophage chemotactic protein-1, macrophage colony-stimulating factor (M-CSF), monokine induced by gamma interferon (MIG), macrophage inflammatory protein-1 (MIP)α, MIP-1β, MIP-2, regulated on activation normal T cell expressed and secreted (RANTES), TNF-α, vascular endothelial growth factor (VEGF).

### Peritoneal macrophage preparation and polarization

Eight to 12-week-old female WT and *Cryab*^*−/−*^ mice were injected intraperitoneally with 3 mL of thioglycollate broth (BD Biosciences). After 3 days, the mice were sacrificed and injected intraperitoneally with 5 mL ice-cold Dulbecco’s modified eagle media (DMEM) (Invitrogen). The DMEM solution within the peritoneal cavity was collected and centrifuged at 1400 rpm for 10 min at 4 °C. The pellet was resuspended in media consisting of 1% pyruvate, 1% glutamine, and 1% penicillin streptomycin in DMEM. Cells were plated at a concentration of 1,000,000 cells/mL at 37 °C in 5% CO_2_. After 4 days, cells were stimulated with or without 100 ng/mL LPS, 100 ng/mL LPS + 100 U/mL recombinant mouse (rm)-IFN-γ (Gibco, PMC4031), or 10 ng/mL rm-IL-4 (Peprotech, 214-14) + 10 ng/mL rm-IL-13 (Peprotech, 210-13), in the presence or absence of 2 μg/mL CRYAB peptide (73-92) [35] (Stanford University Pan Facility, Stanford, California, USA) for 24 or 48 h.

### ELISA

Supernatants from cultured WT and *Cryab*^−/−^ peritoneal macrophages were analyzed for levels of IL-6 (BD Biosciences, 555240), IL-1β (BD Biosciences, 559603), IL-12p40 (BD Biosciences, 555165), and TNF-α (R&D Systems, DY410) by ELISA according to the manufacturer’s instructions.

#### ELISA quantification

The optical density of each well was quantified using the MMP6 software. Briefly, protein content was determined with a spectrophotometer using absorption at 450 nm to analyze the protein concentration as a function of the optical density. Protein concentrations were then calculated using the corresponding standard curves.

### RT-PCR

Two hundred nanograms of RNA was used for reverse transcription into cDNA. The Qiagen QuantiTect Reverse Transcription kit was used, which consists of a gDNA elimination step followed by reverse transcription. After completion of reverse transcription, 65 μL of RNase/DNase free water was added to each sample. qPCR was performed in triplicates using the QuantiFast SYBR Green PCR kit with QuantiTect Primer Assay primers for the following genes: GAPDH, Mm_Gapdh_3_SG; IL-10, Mm_Il10_1_SG; IL-12, Mm_Il12a_1_SG; iNOS, Mm_LOC673161_1_SG; Arg1, Mm_Arg1_1_SG.

### Western blotting

Total protein was isolated from cultured peritoneal macrophages in a solution containing 50 mM Tris-HCL pH 7.4, 1% NP-40, 10% glycerol, 1 mM EDTA, 1 mM Na_3_VO_4_, 1 mM NaF, 1 mM DTT, 4.5 mM sodium pyrophosphate, 10 mM β-glycerophosphate, and a protease inhibitor cocktail tablet (Roche Diagnostics). The supernatants were collected after centrifugation at 14,000 rpm at 4 °C for 20 min, and protein content determined with a spectrophotometer using absorption at 570 nm. Forty micrograms of protein was suspended in two volumes of double-strength sodium dodecyl sulfate (SDS) sample buffer (Bio-Rad Laboratories) and subjected to 6–15% SDS–polyacrylamide gel electrophoresis. Proteins were then transferred to polyvinylidene fluoride membranes that were blocked with 10% non-fat dried milk in Tris–HCl-buffered saline containing 0.05% Tween-20. Membranes were immunoblotted overnight at 4 °C with the following primary antibodies: rabbit anti-CD80 (Abcam, ab53003, 1:2000), rabbit anti-CD86 (Abcam, ab53004, 1:2000), and rabbit anti-actin (Sigma, A2006, 1:1000). Bound primary antibodies were visualized using horseradish peroxidase-conjugated anti-rabbit IgG (GE Healthcare, 1:5000) followed by chemiluminescence detection using an ECL kit (Pierce).

#### Western blot densitometric quantification

Western blot bands were quantified using the ImageJ software. Briefly, arbitrary pixel units were obtained for the optical density (OD) of an area around each band, and a ratio of OD:area was calculated. The OD:area values for a protein of interest were then normalized to the corresponding actin OD:area numbers.

### Statistical analysis

Statistical analysis was performed using Graphpad Prism 6. Data are presented as means ± sem. Data was analyzed using two-tailed independent Student’s *t*-test to detect between-group differences, and two-tailed paired *t*-test to detect differences before and after treatment with CRYAB peptide; *p* < 0.05 was considered significant.

## Results

### *Cryab*^*−/−*^ mice display prolonged presence of macrophages in injured distal sciatic nerves

**Fig. 1:**
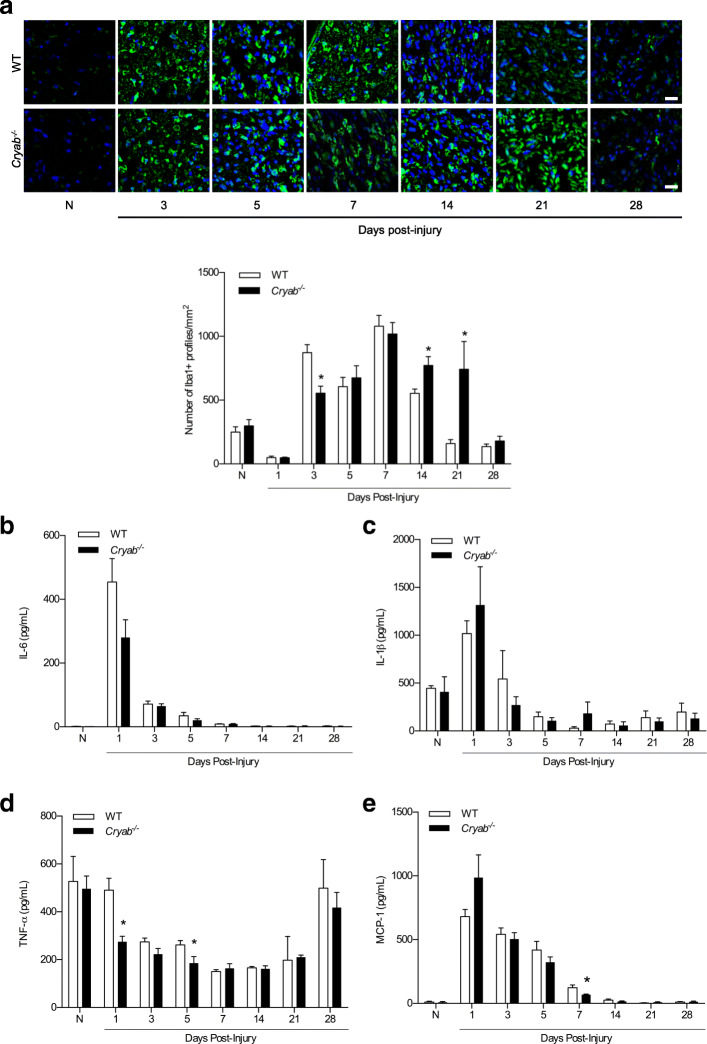
Number of macrophages and level of cytokines/chemokines in crushed sciatic nerves from WT and *Cryab*^−/−^ animals. **a** Representative images and quantification of the number of Iba1^+^ profiles in the sciatic nerves of naïve (N), and 1, 3, 5, 7, 14, 21, and 28 days post-crushed WT (white bars) and *Cryab*^−/−^ (black bars) animals; representative of 2 experiments, *n* = 3–5 animals/group, magnification = × 20, bar = 20 μm, data represent mean ± sem, *p < 0.05 independent *t*-test. **b**–**e** Concentration of IL-6 (**a**), IL-1β (**b**), TNF-α (**c**) and MCP-1 (**d**) in the distal sciatic nerve segments of naïve (N), and 1, 3, 5, 7, 14, 21, and 28 days post-crushed WT (white bars) and *Cryab*^−/−^ (black bars) mice. Representative of 2 experiments, *n *= 3–4 mice/group. Data represent mean ± sem, *p < 0.05 independent *t*-test

CRYAB has been shown to possess immunosuppressive properties in stroke and a model of multiple sclerosis. Because prolonged presence of macrophages and their secretory products can contribute to pain hypersensitivity in damaged peripheral nerves [13–18], we asked whether the enhanced sensation to painful stimuli observed in *Cryab*^*−/−*^ mice with sciatic nerve crush injury [30] may be related to enhanced influx and/or presence of macrophages. As such, we quantified the number of Iba1^+^ DAPI^+^ macrophages in uninjured sciatic nerve at 1, 3, 5, 7, 14, 21, and 28 days after crush damage in WT and *Cryab*^*−/−*^ mice. Immunofluorescence labeling revealed no difference in Iba1^+^ DAPI^+^ counts between uninjured WT (58.83 ± 9.96) and *Cryab*^*−/−*^ (74.40 ± 13.75) nerves (Fig. 1a). At 3 mm distal to a crush injury, there was a marked, expected increase in the number of Iba1^+^ DAPI^+^ macrophages at 3, 5, and 7 days post-damage in WT sciatic nerves relative to uninjured nerves (Fig. 1a). At these time points, *Cryab*^*−/−*^ nerves also showed an increase in the number of Iba1^+^ macrophages similar to that as the WT cohort except at 3 days post-crush where fewer Iba1^+^ DAPI^+^ profiles were observed. At later time points post-damage in WT mice (14 days and onwards), the number of Iba1^+^ DAPI^+^ macrophages decreased precipitously and returned to baseline status by day 21. In injured *Cryab*^*−/−*^ animals, however, the number of Iba1^+^ DAPI^+^ macrophages remained elevated at 14 [193.00 ± 22.05 (*Cryab*^*−/−*^) vs 138.40 ± 9.45 (WT)] and 21 days [185.40 ± 60.94 (*Cryab*^*−/−*^) vs 39.80 ± 8.69 (WT)] post-injury compared to WT mice, and only decreased back to baseline at 28 days post-damage (Fig. 1a). The null animals thus displayed a prolonged presence of macrophages following PNS crush damage.

The similar number of macrophages early after injury (5 and 7 days) between the WT and *Cryab*^*−/−*^ animals (Fig. 1a) suggested that the recruitment of monocytes was not impacted by the small heat shock protein. Indeed, when nerve protein lysates were assayed for the presence of cytokines (IL-6, IL-1β, and TNF-α) and chemokines (MCP-1) known to mediate infiltration of monocytes into damaged peripheral nerves [10, 15, 42], we found no overall difference in their levels between WT and *Cryab*^*−/−*^ mice at days 1–7 post-injury; lower levels of TNF-α were detected at days 1 and 5 in damaged *Cryab*^*−/−*^ nerves (Fig. 1 b–e). At later time points post-injury, the levels of IL-6, IL-1β, TNF-α, and MCP-1 were also indistinguishable between WT and *Cryab* null nerves indicating that although the presence of macrophages was prolonged in *Cryab*^*−/−*^ mice, the cytokine/chemokine content within the nerve was similar to that of WT mice. Other cytokines and chemokines on the luminex array displayed no overall difference (eotaxin, G-CSF, IL-1α, IL-9, IP-10, KC, LIF, MIG, MIP-1α, MIP-1β, MIP-2) or were not detectable (GM-CSF, IFN-γ, IL-2, IL-3, IL-4, IL-5, IL-7, IL-10, IL-12p40, IL-12p70, IL-13, IL-15, IL-17A, LIX, RANTES, VEGF) (data not shown).

### Myelin clearance after nerve injury is not impacted by CRYAB

**Fig. 2:**
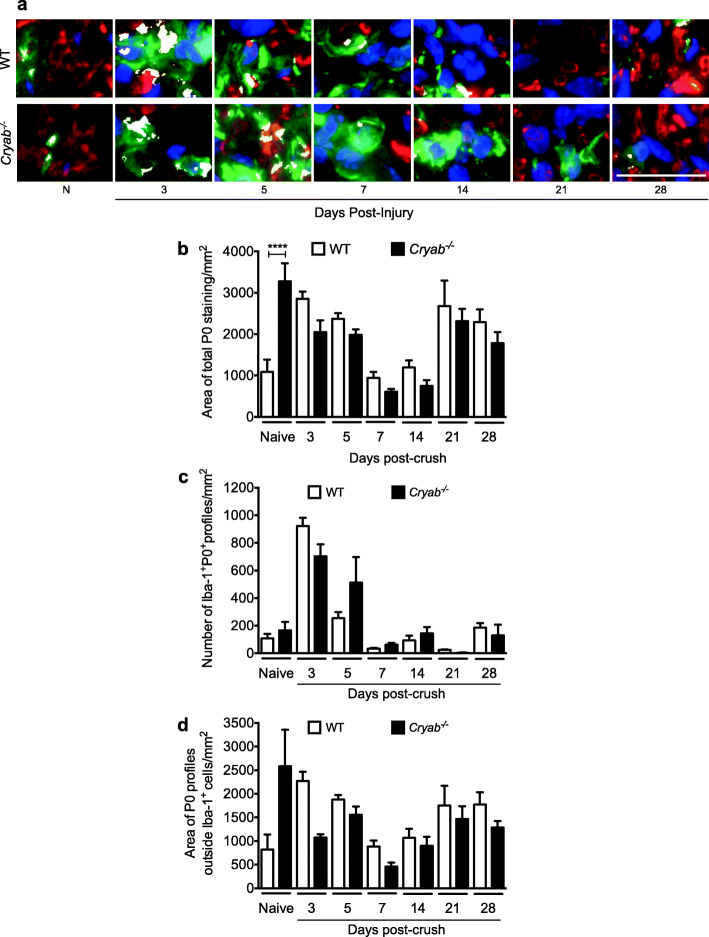
Presence of P0^+^ myelin within and outside of Iba1^+^ DAPI^+^ cells. **a** Representative images of colocalization (white) of Iba1^+^ cells (green), P0^+^ staining (red), and DAPI^+^ (blue) in sciatic nerves from naïve and 3, 5, 7, 14, 21, and 28 days post-crushed WT and *Cryab*^−/−^ mice, bar = 20 μm. **b**, **c** Quantification of the total area of P0 staining (**b**), number of Iba1^+^ DAPI^+^ P0^+^ profiles (**c**), and area of P0 staining outside of Iba1^+^ DAPI^+^ cells (**d**) in sciatic nerves from naïve and injured WT (white bars) and *Cryab*^−/−^ (black bars) animals; *n*=3–7 animals/group, data represent mean ± sem, *p< 0.05 independent *t*-test

To investigate if aberrations in Wallerian degeneration processes may account for the sustained presence of Iba1^+^ cells in damaged *Cryab*^*−/−*^ sciatic nerves, we monitored for macrophage phagocytosis by measuring for the number of Iba1^+^ DAPI^+^ profiles that were protein zero positive (P0^+^). We also measured for total myelin and the amount of P0 staining outside of Iba1^+^ DAPI^+^ cells (Fig. 2). First, as expected after injury in WT animals, the amount of total stained myelin increased in the nerves of WT animals at days 3 and 5 post-damage likely because of unraveling and breakdown of the myelin sheath. This was followed by a decrease at days 7 and 14 after nerve crush that is probably due to myelin debris phagocytosis and digestion by macrophages and Schwann cells. The myelin level then rebounded at day 21 when re-myelination had likely initiated (Fig. 2 a, b). When the amount of total myelin was measured in the injured *Cryab*^*−/−*^ mice, there was no difference relative to the WT controls at any of the time points post-injury, except in the naïve situation where the null animals displayed a higher amount of P0 staining. A similar result was seen when the number of Iba1^+^ DAPI^+^ P0^+^ profiles (Fig. 2 a, c) or amount of myelin outside of Iba1^+^ DAPI^+^ profiles (Fig. 2 a, d) was measured in WT and *Cryab*^*−/−*^ animals. That is, no differences were evident between the two genotypes at any timepoint after damage in the number of Iba1^+^ DAPI^+^ cells that co-stained for P0 or in the amount of P0 profiles outside of Iba1^+^ DAPI^+^ cells. This suggests that the phagocytic ability of macrophages is not dependent on CRYAB.

### The increased number of macrophages in injured *Cryab*^*−/−*^ sciatic nerves is primarily of the pro-inflammatory phenotype

**Fig. 3:**
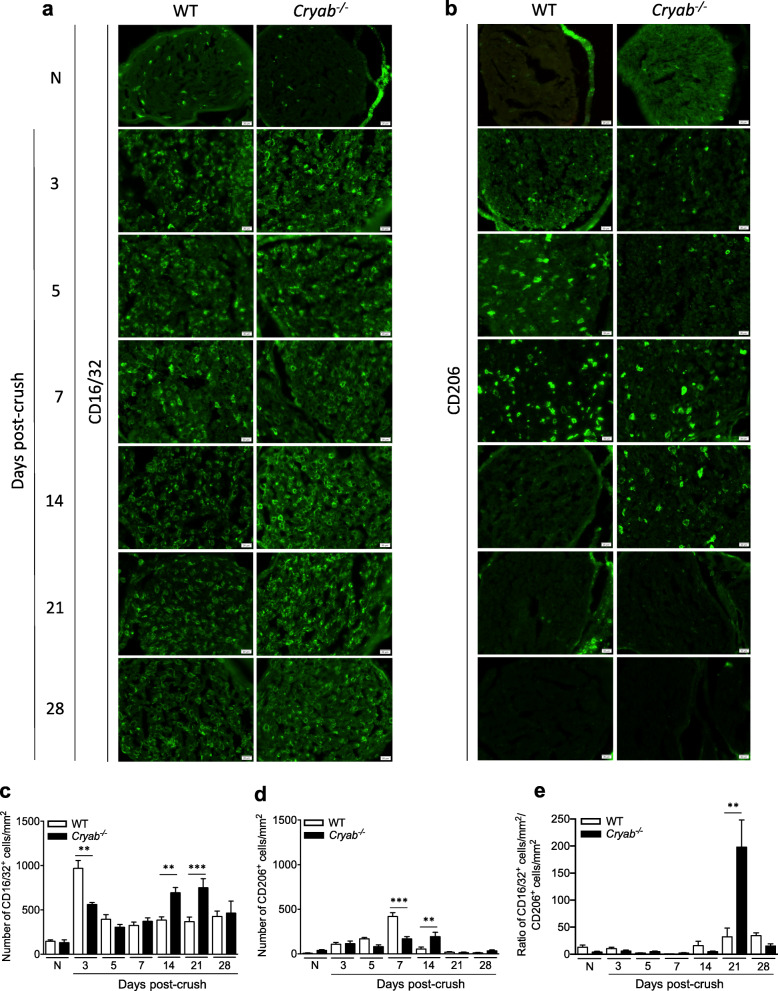
Quantification of the number of pro-inflammatory and immunosuppressive macrophages in WT and *Cryab*^−/−^ crushed sciatic nerves. Representative images and quantification of the number of CD16/32^+^ Iba1^+^ DAPI^+^ (**a**, **c**) and CD206^+^ Iba1^+^ DAPI^+^ (**b**, **d**) profiles in the sciatic nerves of naïve (N), and 3, 5, 7, 14, 21, and 28 days post-crushed WT (white bars) and *Cryab*^−/−^ (black bars) animals; representative of 2 experiments, *n*=3–4 animals/group, bar = 20 μm, data represent mean ± sem, *p< 0.05 independent *t*-test. **e** Graph displaying the ratio of CD16/32^+^ cells to CD206^+^ cells for WT and *Cryab*^−*/*−^ nerves

Since macrophage recruitment (Fig. 1) and phagocytosis (Fig. 2) appeared to be normal between WT and *Cryab*^*−/−*^ animals, we then asked if the prolonged presence of Iba1^+^ cells in the null animals was related to the phenotype of the macrophages. The reason is, macrophages are influenced by the microenvironment during Wallerian degeneration and can exhibit differing functions due to polarization to specific phenotypes [43]. For instance, pro-inflammatory macrophages are mainly present in the early stages after peripheral nerve injury and function in sterilizing wounds and secreting pro-inflammatory cytokines, proteolytic enzymes, and free radicals [8, 19, 44, 45]. Anti-inflammatory macrophages on the other hand, typically balance the pro-inflammatory cells by downregulating inflammation and initiating wound repair at later stages of recovery [8, 19, 44–46]. Therefore, we assessed for the number of Iba1^+^ macrophages that was CD16/32^+^ (pro-inflammatory marker) and CD206^+^ (anti-inflammatory marker) [47] in uninjured and crushed sciatic nerves. For WT animals, the number of CD16/32^+^ Iba1^+^ DAPI^+^ macrophages increased dramatically within 3 days of injury before reducing to a medium level of inflammation from days 5–28 post-injury as compared to naïve animals (Fig. 3a). For *Cryab* null nerves, there was also a significant increase in the number of CD16/32^+^ Iba1^+^ DAPI^+^ cells at day 3 post-damage that remained elevated from days 5–28 (Fig. 3a). Of particular note, the *Cryab*^*−*/*−*^ CD16/32^+^ Iba1^+^ DAPI^+^ counts were significantly lower at day 3 and higher at days 14 and 21 post-damage relative to their WT counterparts, similar to what we observed for Iba1 counts only (Fig. 1a). Expression of CD68 (Fig. S1a) and iNOS (Fig. S1b) in Iba1^+^ cells clarified that these cells were in an activated state.

When the ratio of CD16/32^+^ cells to CD206^+^ cells was analyzed between WT and *Cryab*^*−/−*^ nerves at various time points post-injury (Fig. 3e), the ratio for WT nerves remained similar to naïve levels except for a small increase at days 21 and 28. A similar pattern was seen for null nerves except for a marked increase in the ratio at day 21 post-injury. In comparing the ratio between genotypes, the values were similar between WT and null nerves from days 3 to 7 after damage. At days 14 and 28 after crush, there was a small reduction in the ratio in null nerves relative to WT. At day 21, this pattern was reversed with a marked increase in the *Cryab*^*−/−*^ nerves relative to WT animals.

In regard to anti-inflammatory macrophages, although fewer in numbers compared to CD16/32^+^ Iba1^+^ DAPI^+^ cells, the number of CD206^+^ Iba1^+^ DAPI^+^ macrophages in injured WT sciatic nerves increased progressively from days 3 to 7 post-injury before dropping markedly to baseline counts from day 14 onwards (Fig. 3b). The *Cryab*^*−/−*^ mice also displayed an increase in the number of CD206^+^ Iba1^+^ cells from day 3 after damage but was maintained longer than WT nerves (until day 14 post-crush) before returning to naïve levels at day 21. Significantly fewer and higher numbers of CD206^+^ Iba1^+^ macrophages were noted in the *Cryab*^*−/−*^ nerves at days 7 and 14 respectively compared to WT mice (Fig. 3b). Staining with the immunosuppressive marker, Arg1, verified that some of the Iba1^+^ cells were of an anti-inflammatory nature (Fig. S1c). Thus altogether, pro-inflammatory macrophages were present longer in the peripheral nerves of *Cryab* null animals following PNS crush damage.

### CRYAB suppresses pro-inflammatory cytokine levels in peritoneal macrophages

To try and understand what could be the consequence of prolonged presence of pro-inflammatory macrophages in peripheral nerves after damage, we assessed for the effect of CRYAB on the activation of macrophages. We were specifically interested in pro-inflammatory cytokines since they are involved in neuropathic pain after PNS damage, a pathologic sign that we had previously noted in injured *Cryab*^*−/−*^ mice [30]. Because we saw no difference in the cytokine/chemokine levels between WT and *Cryab*^*−/−*^ uninjured and damaged nerves at late time points post-injury when enhanced macrophages were noted in the *Cryab* null mice (Fig. 1), we asked whether the cytokine/chemokine production by null macrophages could have indeed been altered, but because of other cells in the milieu, e.g., Schwann cells and endothelial cells that may be secreting these factors, this resulted in similar overall levels between WT and *Cryab*^*−/−*^ animals. As such, we moved to an in vitro  paradigm to assess for cytokine production and co-stimulatory molecule expression by WT and *Cryab*^*−/−*^ macrophages that were grown with or without a CRYAB peptide; this peptide has been shown to possess immunomodulatory functions [35]. Also, because Schwann cells and endothelial cells can also secrete cytokines, the culture system allowed us to focus on the responses specific to macrophages.

**Fig. 4:**
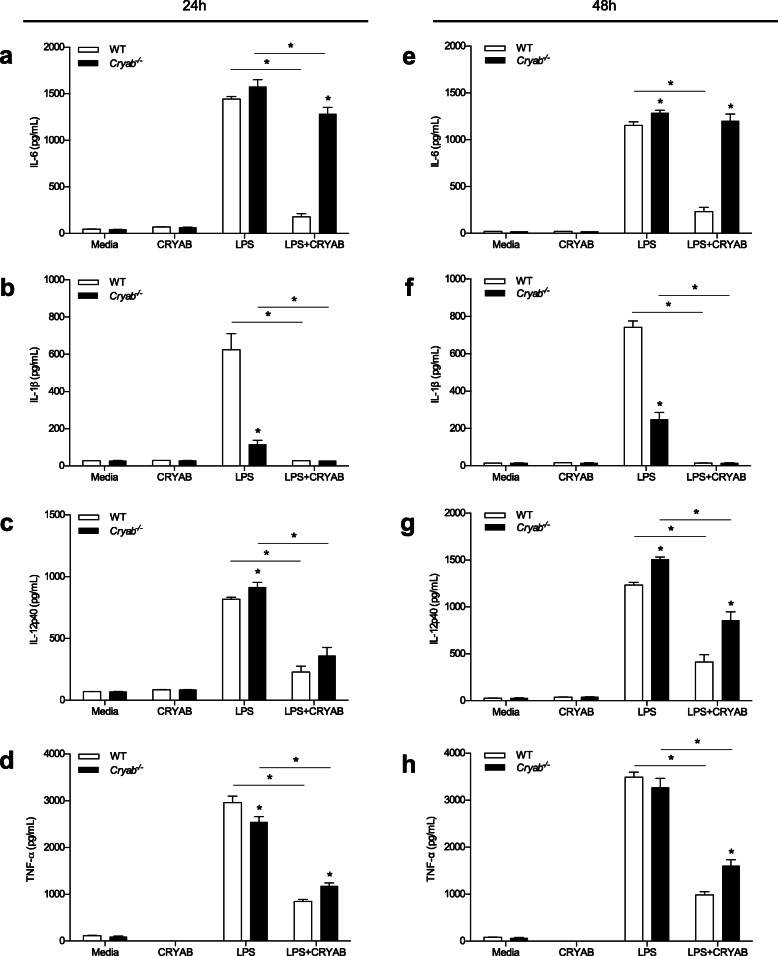
Levels of pro-inflammatory cytokines secreted by macrophages following stimulation with LPS in the presence or absence of CRYAB. Concentration of IL-6 (**a**, **e**), IL-1β (**b**, **f**), IL-12p40 (**c**, **g**), and TNF-α (**d**, **h**) secreted by peritoneal macrophages from WT (white bars) and *Cryab*^−/−^ (black bars) animals following 24 h (**a**–**d**) and 48 h (**e**–**h**) stimulations with LPS and treatment with a CRYAB peptide; representative of 2 experiments for 24 h and 3 experiments for 48 h, *n*=4 mice/genotype with 6 wells/group. Data represent mean ± sem, *p< 0.05 independent *t*-test

Little to no secretion of the pro-inflammatory cytokines IL-1β, IL-6, IL-12p40, or TNF-α was detected in macrophages grown in media alone or CRYAB peptide alone groups from either WT or *Cryab*^*−/−*^ mice (Fig. 4). As expected following LPS stimulation, WT macrophages produced high levels of IL-6, IL-1β, IL-12p40, and TNF-α after 24 (Fig. 4 a–d) and 48 h (Fig. 4 e–h) of culturing. Of note, treating LPS-activated peritoneal WT macrophages with the CRYAB peptide significantly reduced the amount of IL-6, IL-1β, IL-12p40, and TNF-α secreted at 24 (Fig. 4 a–d) and 48 h (Fig. 4 e–h) post-stimulation.

Similar to WT macrophages after LPS stimulation, cells from *Cryab*^*−/−*^ mice also secreted high levels of IL-6, IL-1β, IL-12p40, and TNF-α after 24 (Fig. 4 a–d) and 48 h (Fig. 4 e–h) of stimulation. Notably, the amount of IL-6 and IL-12p40 produced by the LPS-activated *Cryab*^*−/−*^ macrophages was significantly higher than WT levels at both time points whereas lower amounts of IL-1β were noted in the *Cryab* null macrophages with no overall changes seen for TNF-α (Fig. 4 a–h). Following CRYAB peptide treatment of *Cryab*^−/−^ macrophages, we observed a similar effect as that seen for WT cells, where IL-1β, IL-12p40, and TNF-α production was reduced after 24 h (Fig. 4 b–d) and 48 h (Fig. 4 f–h). Interestingly, the difference in cytokine production between WT and *Cryab*^−/−^ macrophages was more pronounced after CRYAB peptide application with null macrophages exhibiting higher levels of IL-6 and TNF-α production after 24 h (Fig. 4 a, d), and greater levels of IL-6, IL-12p40, and TNF-α secretion after 48 h (Fig. 4 e, g, h) compared to treated WT macrophages. Taken together, these data suggest that in vitro, CRYAB has the ability to limit the amount of pro-inflammatory cytokines secreted by macrophages and that its removal augments production of such cytokines.

### CRYAB does not modulate the machinery for antigen presentation

**Fig. 5:**
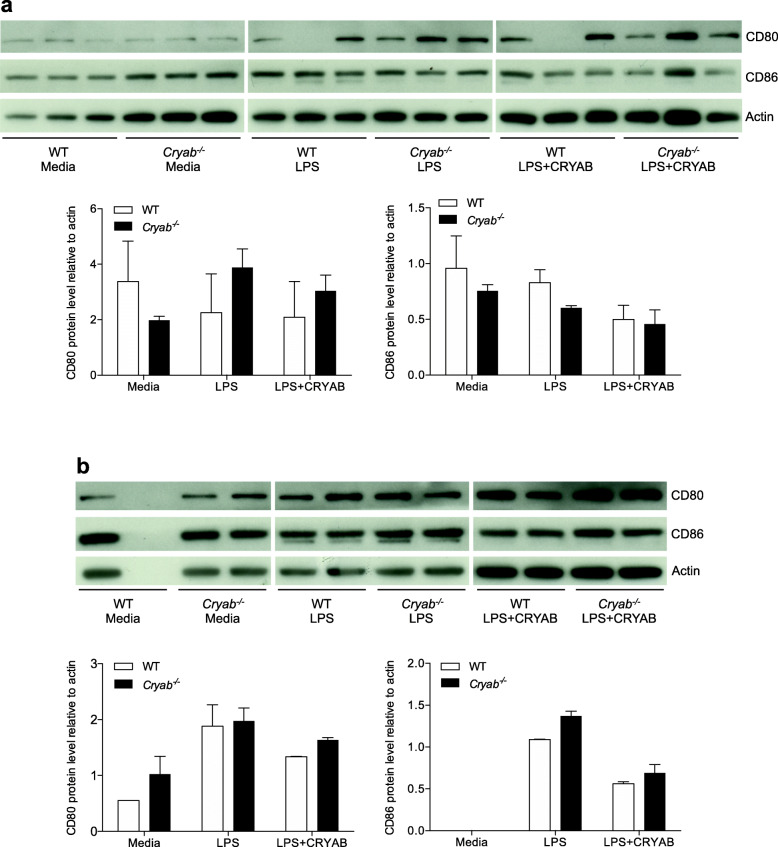
Levels of CD80 and CD86 following LPS stimulation with or without CRYAB. Western blot levels and ImageJ analysis of CD80 and CD86 in macrophages from WT (white bars) and *Cryab*^−/−^ (black bars) animals cultured in media, LPS, or LPS with CRYAB peptide for 24 h (**a**) or 48 h (**b**); blots are representative of 3 experiments, graphs display accumulated data from 3 experiments where *n*=2–3 mice/group/experiment. Data represent mean ± sem, *p< 0.05 independent *t*-test

In addition to secreting cytokines, activated macrophages act as antigen presenting cells to trigger adaptive immunity and to activate helper T-cells [48] where the co-stimulatory molecules, CD80 and CD86, play a crucial role in initiating and maintaining an immune response through antigen presentation [49]. We therefore assessed if the expression of CD80 and CD86 in peritoneal macrophages was altered by CRYAB. No difference in the expression levels of CD80 and CD86 was observed between WT and *Cryab*^−/−^ macrophages following stimulation with LPS for 24 h (Fig. 5a) or 48 h (Fig. 5b). Further, treatment with CRYAB peptide did not alter the levels of CD80 and CD86 after 24 h (Fig. 5a) or 48 h (Fig. 5b) for either WT or *Cryab*^−*/*−^ macrophages. These results suggest that CRYAB does not alter the antigen presenting capabilities of macrophages.

### Cytokine production by cultured pro-inflammatory polarized peritoneal macrophages is reduced by CRYAB

**Fig. 6:**
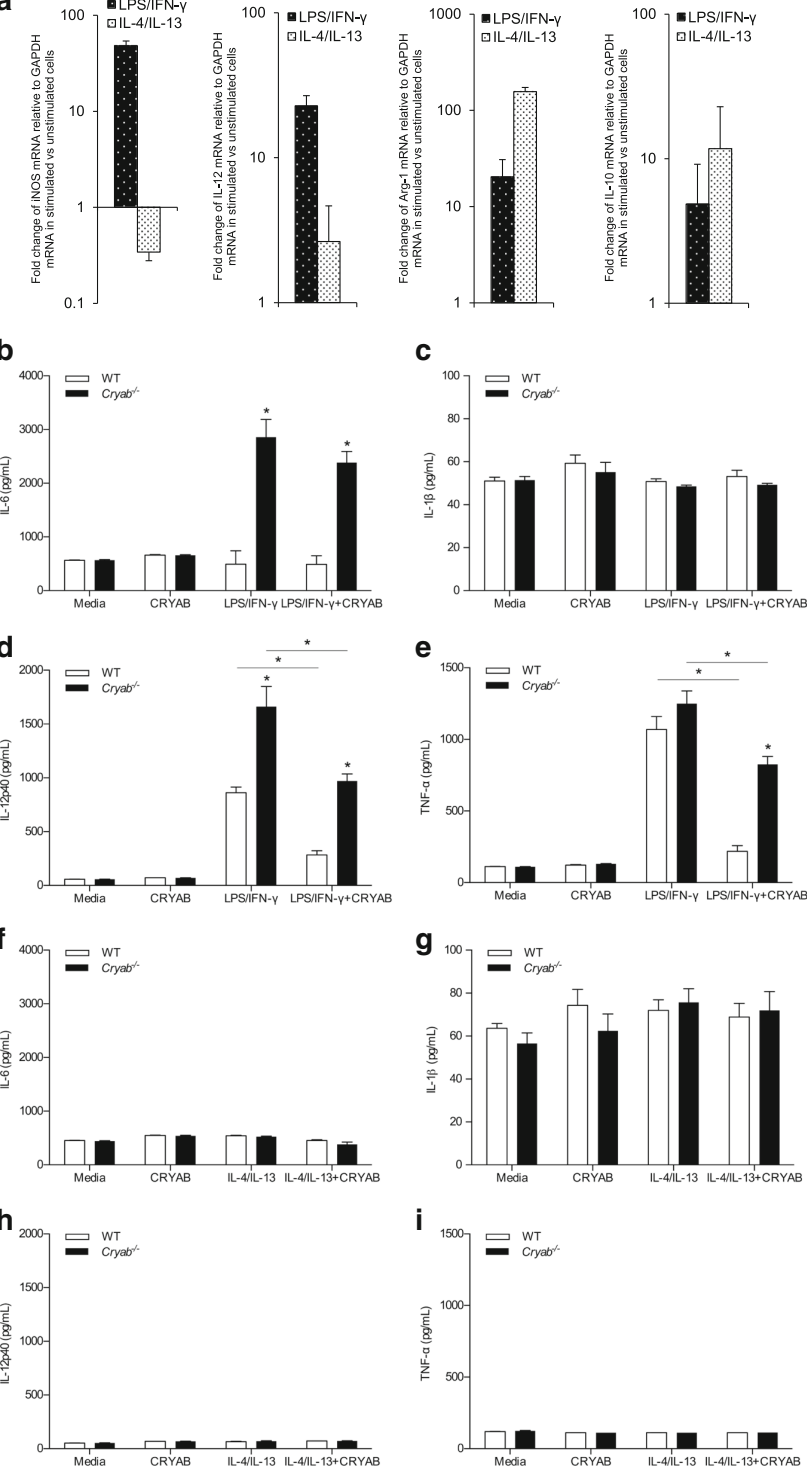
Secretion of cytokines by pro-inflammatory^LPS/IFN-γ^ and anti-inflammatory^IL-4/IL-13^-polarized macrophages following treatment with CRYAB. **a** mRNA levels of iNOS, IL-12p40, arginase-1, and IL-10 in LPS/IFN-γ- and IL-4/IL-13-polarized macrophages; representative of 3 experiments, *n*=3–4 wells/group. **b**–**i** Concentration of IL-6 (**b**, **f**), IL-1β (**c**, **g**), IL-12p40 (**d**, **h**), and TNF-α (**e**, **i**) secreted by pro-inflammatory^LPS/IFN-γ^ (**b**–**e**) and anti-inflammatory^IL-4/IL-13^ (**f**–**i**) -polarized macrophages from WT (white bars) and *Cryab*^−/−^ (black bars) animals grown for 48 h in the presence or absence of a CRYAB peptide; representative of 3 experiments, *n*=4 wells/group. Data represent mean ± sem, *p< 0.05 independent *t*-test

Finally, we interrogated whether the anti-inflammatory effects of CRYAB on macrophage presence in injured sciatic nerves (Fig. 1a) and cytokine production in vitro (Fig. 4) depended on the phenotype of the macrophages. To this end, we once again employed the peritoneal macrophage in vitro paradigm. Using a combination of LPS and IFN-γ, macrophages were polarized towards a pro-inflammatory phenotype as identified by high iNOS and IL-12p40 mRNA expression, and low levels of arginase-1 and IL-10 mRNA (Fig. 6a) while polarization of macrophages toward an anti-inflammatory phenotype was achieved by using a combination of IL-4 and IL-13 (Fig. 6a). These latter cells displayed high amounts of arginase-1 and IL-10 mRNA and low levels of iNOS and IL-12p40 (Fig. 6a).

Stimulation of WT macrophages for 48 h with LPS and IFN-γ resulted in an increase in the secretion of the pro-inflammatory cytokines IL-6, IL-12p40, and TNF-α (Fig. 6 b, d, e). Activation of *Cryab*^−/−^ macrophages with LPS and IFN-γ also resulted in an increase in IL-6, IL-12p40, and TNF-α (Fig. 6 b, d, e) with IL-6 and IL-12p40 being produced at much higher levels relative to their WT counterparts (Fig. 6 b, d). The addition of CRYAB peptide for 48 h resulted in significant decreases in the secretion levels of IL-6, IL-12p40, and TNF-α in WT, as well as in *Cryab*  null cells (Fig. 6 b, d, e). Of note, the elevated level of cytokines normally produced by *Cryab*^−/−^ macrophages as compared to WT cells was maintained for IL-12p40 and TNF-α upon CRYAB peptide treatment (Fig. 6 d, e). These data indicate that CRYAB has the ability to modulate the function of pro-inflammatory-polarized macrophages by attenuating their secretion of certain pro-inflammatory cytokines.

As expected, anti-inflammatory-polarized macrophages displayed low levels of IL-6, IL-1β, IL-12p40, and TNF-α in both WT- and *Cryab*^−/−^-derived cells, and addition of a CRYAB peptide did not alter the levels of the cytokines (Fig. 6 f–i). To determine whether the heat shock protein could influence the anti-inflammatory properties of IL-4/IL-13-polarized macrophages, we assessed for IL-10 production, a typical readout for such polarized cells. The levels of IL-10 were inconsistent over three experiments (*n*=4 replicates/experimental group for each separate experiment) and thus no conclusions could be drawn (data not shown). Taken together, these in vitro data suggest that CRYAB may preferentially modulate pro-inflammatory macrophages by decreasing their secretion of pro-inflammatory cytokines.

## Discussion

Inflammation constitutes a double-edged sword with respect to nerve regeneration. Many studies have demonstrated the critical role that macrophages play in successful nerve regeneration where depletion of these immune cells before a peripheral nerve injury resulted in reduced myelin debris clearance, loss of neurotrophin synthesis, and decreased axon regeneration and functional recovery [1, 8–10]. However, macrophages also play a vital role in the development of peripheral nerve injury-induced neuropathic pain [13–18], and hyper-responsive pro-inflammatory macrophages can cause tissue damage, while overactive immunosuppressive macrophages can promote fibrosis and exacerbate cytotoxic and allergic reactions [8]. Therefore, macrophage responses must be carefully controlled to harness their beneficial properties that allow for regeneration while preventing their potentially detrimental effects.

Previous studies have highlighted the protective role of CRYAB in decreasing inflammation. The small heat shock protein was shown to reduce T-cell proliferation [33], decrease the secretion of pro-inflammatory cytokines by pathogenic CD4^+^ T-cells [33, 35], and protect myoblasts from TNF-α-induced cytotoxicity [28]. In the CNS, CRYAB reduced neuroinflammation and motor deficits in an animal model of multiple sclerosis [33], suppressed cytokine production by astrocytes [33, 50], and decreased microglial activation, and recruitment of inflammatory Ly6C^+^ macrophages following spinal cord injury [32]. Due to its expression in PNS axons and Schwann cells [38–40], we investigated whether CRYAB plays a role in macrophage presence after peripheral nerve injury. We found that *Cryab*^−/−^ mice had an elevated number of macrophages in injured distal nerve segments at late timepoints post-damage as compared to WT mice. This increase in Iba1^+^ macrophages was not due to differential macrophage recruitment following peripheral nerve injury since the number of Iba1^+^ cells was comparable between WT and *Cryab*^−/−^ mice during early time points (1, 3, 5, and 7 days) post-crush. As well, the main cytokines and chemokines responsible for the recruitment of macrophages into the injured site after PNS injury, IL-6, IL-1β, TNF-α, and MCP-1, displayed similar expression profiles in both WT and *Cryab*^−/−^ mice indicating that the recruitment of macrophages after PNS injury was not dependent on CRYAB. It also appears that other aspects of Wallerian degeneration such as macrophage phagocytic ability were not responsible for the prolonged presence of the immune cells since no differences were evident between WT and *Cryab*^−*/*−^ mice in P0 presence within and outside of Iba1^+^ cells. Rather, it appears that the heat shock protein may be involved in resolution of the inflammatory response. In regard to the P0 data, we hypothesize that the gradual increase in P0 area from day 14 onwards in Fig. 2a and c is because regenerating axons are being remyelinated after a period of myelin degradation and clearance (days 3–7). In Fig. 2b, we speculate that the number of P0^+^ Iba1^+^ counts declines at days 7–21 because myelin is being digested by these cells. Eventually, at day 21, we suggest that all degraded myelin has been cleared so P0^+^ Iba1^+^ phagocytosing macrophages are no longer evident/needed in the nerve. Once the nerve is fully regenerated and remyelinated at ~day 28, the system goes back to a homeostatic level as in the naïve situation where there is low basal number of Iba1^+^ P0^+^ cells.

One mechanism to control inflammatory responses is to reduce immune cell numbers by either inducing apoptosis or stimulating re-entry into the vascular circulation. The rebound of CRYAB expression we previously observed at late time points following PNS crush injury [30] may be involved in this process of immune cell reduction since macrophages remained prominently elevated in the nerves of injured null animals at days 14 and 21 post-crush, a time when their numbers should return to baseline status (Fig. 1a). In alignment with our results, Klopstein et al. [32] observed a decline in Ly6C^+^ macrophages in injured spinal cord after treating with recombinant human CRYAB. This would be beneficial since Ly6C^+^ macrophages possess phagocytic, proteolytic, and inflammatory functions and have been associated with many pathological conditions [5]. We did note that although macrophage presence remained high at late time points in *Cryab* null mice, the levels of cytokines and chemokines were similar to that of WT animals. It is possible that other cell types present in the nerve such as Schwann cells and endothelial cells which can also secrete these factors [19] may be contributing to the overall levels of these inflammatory factors.

Another mechanism to prevent macrophage-mediated damage is to suppress activation of pro-inflammatory cells. We show in vitro that CRYAB has the ability to attenuate the secretion of pro-inflammatory cytokines, where treatment with a CRYAB peptide resulted in significantly reduced secretion of IL-6, IL-1β, IL-12p40, and TNF-α following LPS stimulation. Consistent with this observation was that *Cryab*^−/−^ cells generally produced higher levels of the pro-inflammatory cytokines compared to WT macrophages even after CRYAB treatment. Our data also indicates that there is selectivity in the actions of CRYAB. The immunosuppressive capabilities of CRYAB appear to be specific to pro-inflammatory-polarized macrophages and not IL-4/IL-13-polarized cells (Fig. 6). As well, the heat shock protein targeted cytokine reduction and not antigen presentation since expression levels of the co-stimulatory molecules CD80 and CD86 were not altered in null macrophages or by application of CRYAB. It should be noted, however, that additional work needs to address if the in vitro data mirrors what occurs in the nerve in vivo. It is also well-known that tissue macrophages vary substantially based on their origin [51] and the peritoneal macrophages we used may not reflect the proliferating endoneurial and infiltrating monocytes/macrophages present within damaged peripheral nerves. Another caveat is that LPS was used as a general macrophage stimulator but it is possible that other stimulants could produce different responses [19]. Our future studies seek to isolate macrophages from the nerves for phenotypic and activation characterization.

A growing number of studies have implicated pro-inflammatory-polarized macrophages in driving the neuroinflammation that causes neuropathic pain after peripheral nerve injury, that is, increased sensitivity to thermal stimulation and mechanical allodynia [13–18]. Depletion of macrophages in diabetic rats limited the progression of neuropathic pain by reducing mechanical allodynia [16], and treatment with etifoxine improved locomotion, motor coordination, and sensory functions by decreasing the number of macrophages [11]. We previously showed that injured *Cryab*^−/−^ mice display heightened sensitivity to thermal and mechanical stimulation [30]. This hypersensitivity could be related to the prolonged presence of pro-inflammatory macrophages we see here since the time window (days 14 and 21) overlaps with the period of increased sensitivity we had noted in *Cryab*^−*/*−^ mice compared to WT counterparts in von Frey Hair examinations (days 8–42 post-crush damage) [30]. Although cytokine and chemokine levels in the nerve were not different between WT and null animals unlike what we saw for macrophages in the in vitro assays we used, it is plausible that the low levels of cytokines could still contribute to pain sensations. However, it should be noted that in addition to macrophages, it is possible that spinal cord microglia may also be involved in the pain signs [52–55].

It is still unclear why macrophage presence was prolonged in the nerves of injured *Cryab*^−/−^ animals. We tested if a defect in Wallerian degeneration processes such as phagocytic ability of the macrophages may be a factor, but we noted no overall difference in the presence or clearance of P0^+^ myelin between the two genotypes. It is possible that factors secreted by other cells in the nerve environment such as Schwann cells and endothelial cells may play a role in maintaining the presence of the immune cells. Interestingly, we had previously shown in injured *Cryab*^−*/*−^ mice that there may be a defect in de-differentiated Schwann cells being able to differentiate back to a (re)myelinating phenotype. That is, there were fewer P0^+^ Schwann cells at days 14 and 21 post-injury [30] which aligns nicely with our macrophage data here. It is possible that differentiated myelinating Schwann cells secrete or express factor(s) needed for clearance of pro-inflammatory macrophages after peripheral nerve injury. On the flip side, it is also possible that initiation of resolution events such as IL-10 production is delayed in the *Cryab* null nerves. We did assess for IL-10 levels in injured peripheral nerves but its levels were below the detection range. Similarly, in our in vitro experiments, the levels of IL-10 were inconsistent, and thus, it is unclear whether this immunosuppressive cytokine is playing a meaningful resolution role in our injury model and in vitro system. Our future studies aim to identify any resolution factors and/or macrophage clearance factors.

## Conclusions

Overall, this study shows that CRYAB is associated with the presence of pro-inflammatory macrophages following peripheral nerve injury and that the heat shock protein may reduce production of cytokines in these immune cells when they are polarized to a pro-inflammatory state. CRYAB could therefore act by decreasing exuberant inflammation after PNS injury.

## Supplementary Information

**Additional file 1: Supplementary Fig. S1.** Immunostaining of Iba1^+^ cells with CD68, iNOS and Arg1. Micrographs of CD68^+^ Iba1^+^ DAPI^+^ (a), iNOS^+^ Iba1^+^ DAPI^+^ (b) and Arg1^+^ Iba1^+^ DAPI^+^ (c) cells in sciatic nerves at 14 days post-injury in WT and *Cryab*^-/-^ mice; bar = 20 μm.

## Data Availability

All data generated or analyzed during this study are included in this published article.

## Abbreviations

DMEM: Dulbecco’s modified eagle media
DTT: Dithiothreitol
EDTA: Ethylenediaminetetraacetic
ELISA: Enzyme-linked immunosorbent assay
G-CSF: Granulocyte-colony-stimulating factor
GM-CSF: Granulocyte-macrophage colony-stimulating factor
IFN: Interferon
IL: Interleukin
IP-10: Interferon gamma-induced protein 10
KC: Keratinocyte chemoattractant
LIF: Leukemia inhibitory factor
LIX: Lipopolysaccharide-inducible CXC chemokine
LPS: Lipopolysaccharide
MCP: Macrophage chemotactic protein
MIG: Monokine induced by gamma interferon
MIP: Macrophage inflammatory protein
NaF: Sodium fluoride
Na_3_VO_4_: Sodium vanadate
OD: Optical density
P0: Protein zero
PNS: Peripheral nervous system
RANTES: Regulated on activation normal T cell expressed and secreted
SDS: Sodium dodecyl sulfate
TNF: Tumor necrosis factor
Tris-HCL: Tris-hydrocloride
VEGF: Vascular endothelial growth factor
WT: Wildtype
