# Supplementary figures and images for: Presence and activation of pro-inflammatory macrophages are associated with CRYAB expression in vitro and after peripheral nerve injury
Source: J Neuroinflammation. 2021 Mar 24;18:82. doi: 10.1186/s12974-021-02108-z (PMC7992798; doi:10.1186/s12974-021-02108-z)

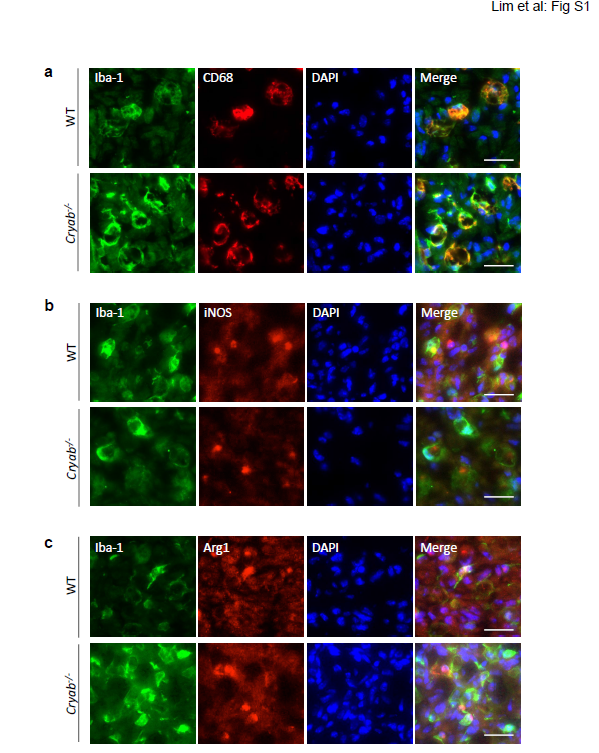

Supplement: Supplementary file 1 — Additional file 1: Supplementary Fig. S1. Immunostaining of Iba1+ cells with CD68, iNOS and Arg1. Micrographs of CD68+ Iba1+ DAPI+ (a), iNOS+ Iba1+ DAPI+ (b) and Arg1+ Iba1+ DAPI+ (c) cells in sciatic nerves at 14 days post-injury in WT and Cryab-/- mice; bar = 20 μm. [file 12974_2021_2108_MOESM1_ESM.docx]
